# The First Non-African Case of *Trichophyton rubrum* var. *raubitschekii* or a Urease-Positive *Trichophyton rubrum* in Central Europe?

**DOI:** 10.1007/s11046-014-9751-7

**Published:** 2014-05-04

**Authors:** Zygmunt Adamski, Michał J. Kowalczyk, Kinga Adamska, Honorata Kubisiak-Rzepczyk, Monika Bowszyc-Dmochowska, Agnieszka Banaszak, Paweł Bartkiewicz, Ryszard Żaba

**Affiliations:** 1 Department of Dermatology, Poznan University of Medical Sciences, Poznan, Poland; 2 Department of Dermatology and Venereology, Poznan University of Medical Sciences, Przybyszewskiego 49, 60-355 Poznan, Poland; 3 Department of Medical Mycology, Poznan University of Medical Sciences, Poznan, Poland; 4 Cutaneous Histopathology and Immunopathology Section, Department of Dermatology, Poznan University of Medical Sciences, Poznan, Poland

**Keywords:** Dermatophytes, Dermatophytosis, Epidemiology, Poland, Itraconazole, Diagnosis, Urease

## Abstract

We report a case of a 34-year-old Polish Caucasian male who was diagnosed with tinea manuum caused by *Trichophyton rubrum* var. *raubitschekii*. It would be the first described case of a dermatophytosis caused by this fungus in Poland and one of a few cases in Central Europe described so far. Admittedly, it would be the first case in Central Europe with no evidence pointing to African origin. The clinical condition improved after administering itraconazole (daily dose 100 mg orally) supplemented with a topical treatment, while the patient was totally cured after 2 months. The histopathological examination turned out to be highly useful in the diagnostic process. The genetic analysis of the urease gene pointed to a urease-positive *T*. *rubrum* rather than *T*. *rubrum* var. *raubitschekii.*

## Introduction

People suffering from fungal infections comprise a substantial group among dermatological patients. Mycoses of the glabrous skin are predominantly triggered by dermatophytes, while members of the *Trichophyton* genus, including *Trichophyton rubrum,* are among the most commonly diagnosed fungal pathogens in Poland. Superficial mycoses resulting from infections by *T*. *rubrum* var. *raubitschekii* are usually found in Africa, Southeast Asia, Australia and South America [1, 2]. In recent years, however, there have been a few cases in Germany and Italy; hence, the pathogen should be considered as undoubtedly present in Europe [3, 4]. To date, there has been no described case of an infection caused by this pathogen in Poland.

## Case Report

**Fig. 1:**
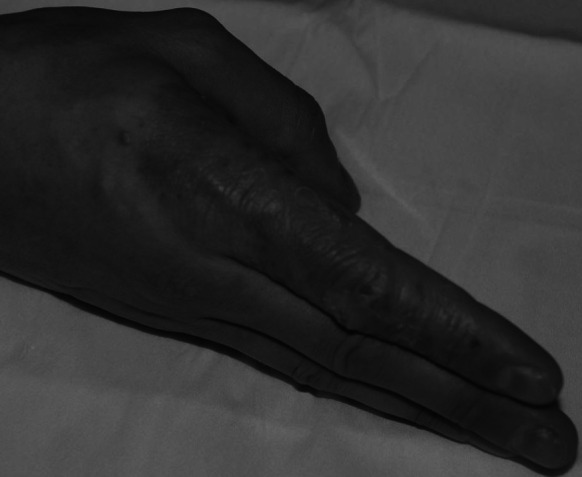
Side of the index finger—hospital admission date

**Fig. 2:**
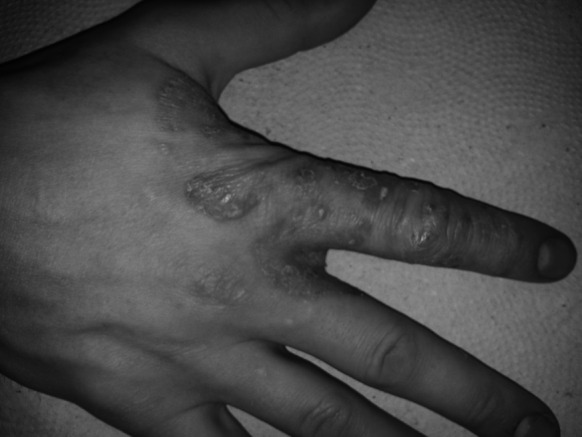
*Top of the right hand*—date of the second sample acquisition during the follow-up appointment at the Department of Medical Mycology, Poznan University of Medical Sciences

A 34-year-old Polish Caucasian male was admitted to the Dermatology Clinic of the Poznan University of Medical Sciences due to the presence of an erythematous, exfoliating, clearly distinct lesion located on the index finger of the right hand, spreading onto surrounding areas (Figs. 1, 2).

The first signs of the disease had appeared in August 2012, after a contact with raw fish—the patient is a cook. Due to a suspicion of erysipeloid, the patient underwent an antibiotic therapy (intravenous clindamycin, 2 × 600 mg daily, for the period of 5 days) coupled with steroids administered topically.

A series of diagnostic examinations were carried out during his hospitalization. A swab for bacteriological, skin biopsy for histopathological and a sample for mycological examinations were acquired. The bacterial culture was found to be positive for *Enterococcus faecalis*. The direct mycological examination was negative. Other examinations, such as the mycological culture and histopathology, were awaited for during the stay of the patient at the clinic. Due to the lack of significant improvement, the patient was discharged, while any further procedures were performed on an outpatient basis at the Dermatology Counseling Service of the clinic.

**Fig. 3:**
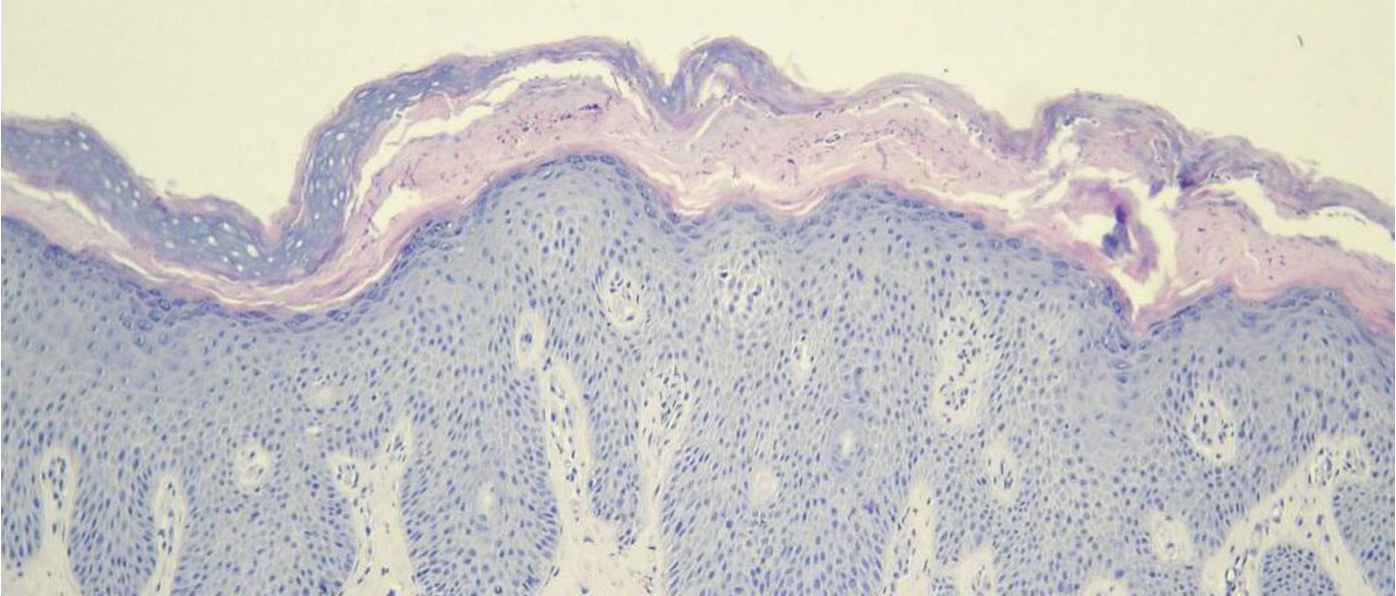
A thickened, swollen epidermis. A thick stratum corneum with numerous pathogenic fungi. Moderate, mixed inflammatory infiltrates present in the dermis. Hematoxylin and eosin stain, objective lens magnification: × 10

**Fig. 4:**
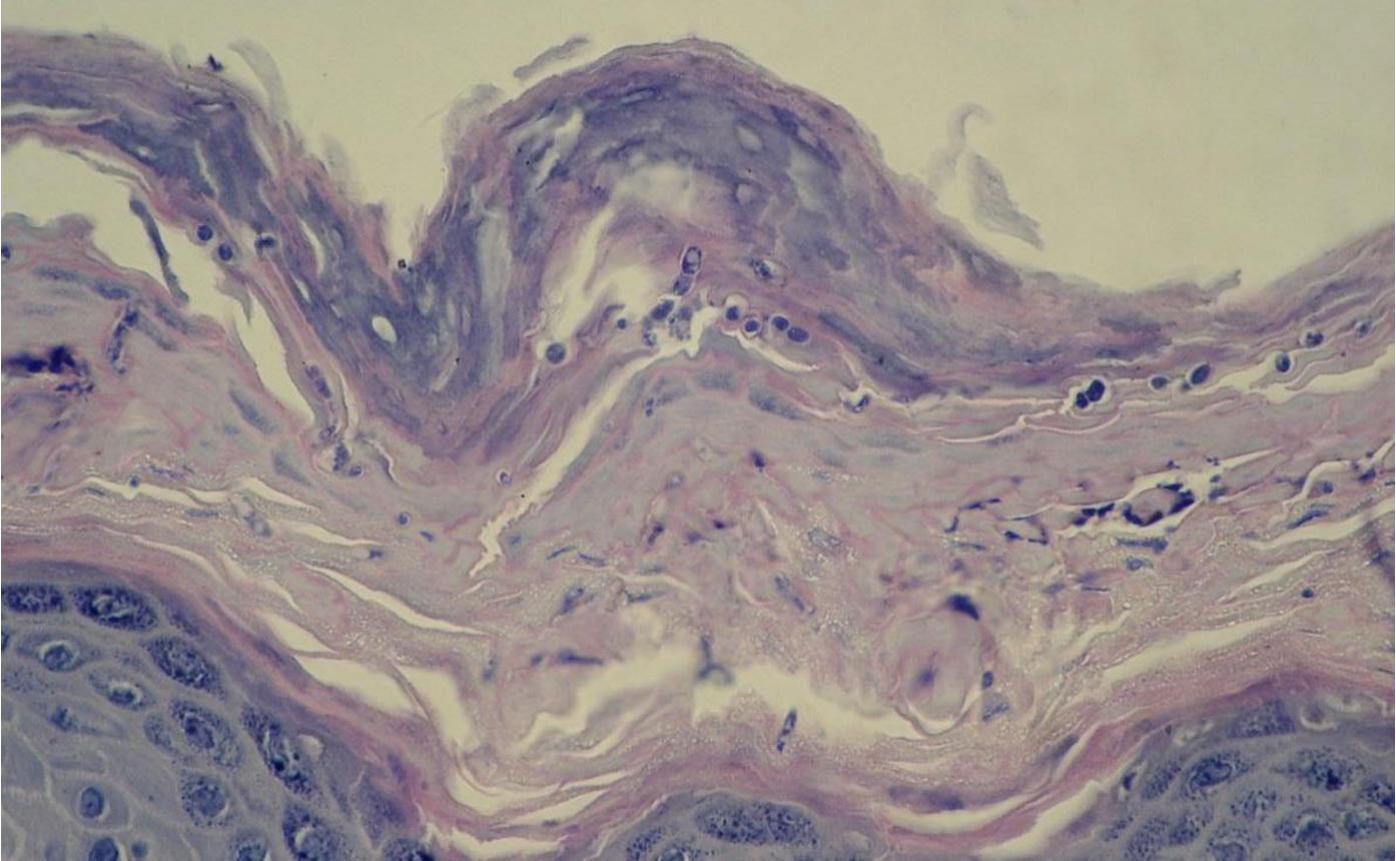
Numerous spores in the stratum corneum. Hematoxylin and eosin stain, objective lens magnification: × 60

The results of the histopathological examination indicated a widened, swollen epidermis with a thickened stratum granulosum and a thick stratum corneum, in which there were numerous, round, fungal spores of 2.5 μm in diameter (Figs. 3, 4). On the other hand, the result of the mycological culture was negative. Due to discrepancies in the results, a second sample for mycological examination was acquired during a follow-up appointment. This time the results of the direct mycological examination were positive.

Itraconazole (daily dose 100 mg) was administered orally. Additionally, an imidazole derivative was administered topically. The patient claimed an improvement in his dermatological condition after 2 weeks.

**Fig. 5:**
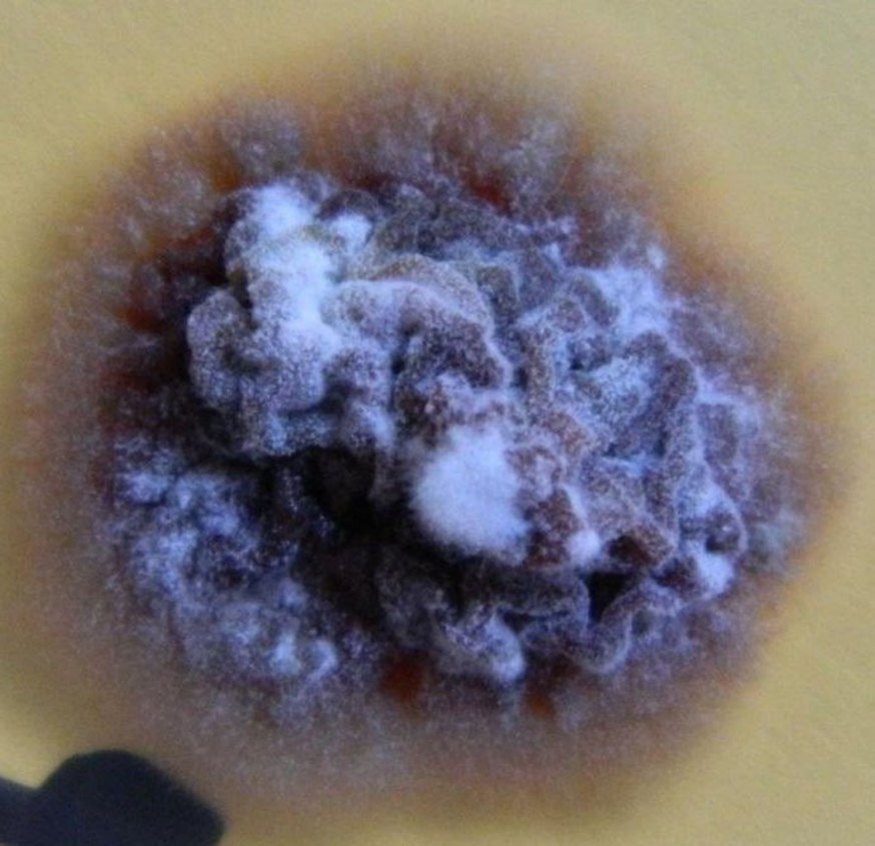
*Trichophyton rubrum* var. *raubitscheki*. A 20-day-old colony grown on Sabouraud agar medium

**Fig. 6:**
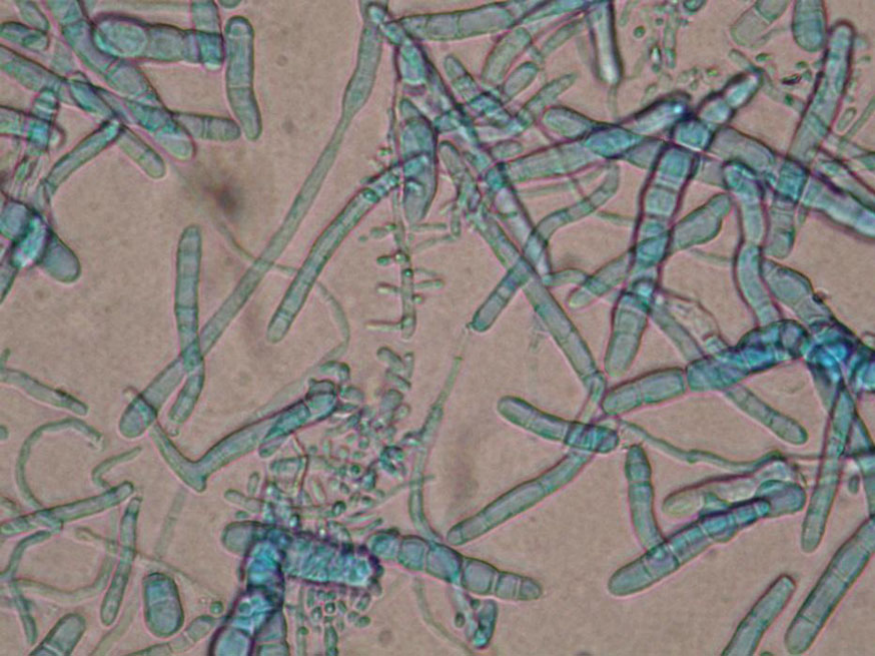
*Trichophyton rubrum* var. *raubitschekii*. Microscopic image (magnification— × 400, lactophenol cotton blue). Numerous macro- and microconidia

The mycological cultures were maintained for 20 days at 25 °C on two kinds of media: Sabo Sabouraud dextrose agar with chloramphenicol and Sabo Sabouraud dextrose agar with chloramphenicol and actidione. A fungal colony with a reddish brown color and a brownish reverse, strongly furrowed, suede-like surface and a raised center was observed (Fig. 5). A microscopic examination revealed numerous cigar-shaped and thin-walled septate macroconidia. Pyriform microconidia were located on short branches along hyphae (Fig. 6). A urease test, carried out on Christiansen’s medium, and dermatophyte test medium examination were both positive. Since the strain matched the original description, it was identified as *T*. *rubrum* var. *raubitschekii* [5].

**Fig. 7:**
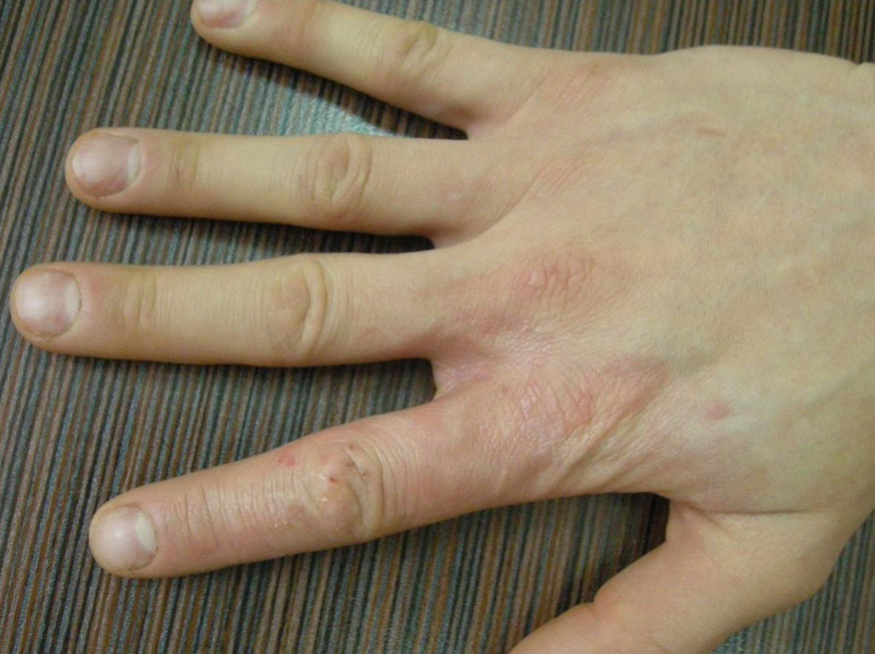
*Top of the right hand*—after 3 weeks of itraconazole treatment

The following 3 weeks brought a further improvement in the dermatological status (Fig. 7). It was decided to continue the itraconazole treatment while the topical drug was switched to a pyridinone derivative. The patient received itraconazole for a period of 2 months, yet due to low tolerance to the topical drug, the pyridinone derivative was no longer administered. Afterward, the patient showed no signs of the infection.

In order to reassess the diagnosis, we analyzed a ribosomal RNA genomic fragment [6] and the urease gene of this isolate as described by Hiruma et al. [7]. Because the isolate ceased to grow on any new media, we isolated its DNA from a plate deposited for long-term storage at our clinic. One hundred and twenty milligrams of the colony was acquired from the deposited agar plate and ground in liquid nitrogen. DNA was isolated with the use of the column-based GeneMATRIX Plant & Fungi DNA Purification Kit (EURx, Poland) that includes both RNase A and proteinase K.

Firstly, we analyzed a fragment containing partial 18S rRNA gene, ITS1, 5.8S rRNA gene, ITS2 and partial 28S rRNA gene with the use of common ITS1 and ITS4 primers for PCR and sequencing. PCR was performed with the following ingredients: 25 pmol of ITS1 and ITS4 primers each [6], 0.5 µl of FastStart Taq DNA Polymerase (2.5 U, Roche), 10 µl of 5 × GC-rich solution, 5 µl of 10 × PCR buffer with 20 mM MgCl_2_, 1 µl of dNTPs (10 mM each), 5 ng of DNA sample, all in a volume of 50 µl. PCR was performed with the following program: pre-denaturation—4 min, 95 °C; amplification—35 × [denaturation: 1 min, 95 °C; annealing: 1 min, 55 °C; elongation: 1 min, 72 °C]; cooling: 1 min, 40 °C. Gel electrophoresis and paired-end sequencing revealed a 692-bp-long fragment, presenting a 100 % coverage with multiple GenBank *T*. *rubrum* entries and with our own *T*. *rubrum* reference strain. This result was expected as *T*. *rubrum* and *T*. *rubrum* var. *raubitschekii* were found to be identical in this genomic region [8].

**Fig. 8:**
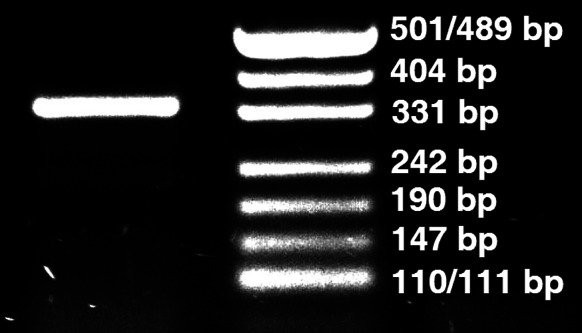
Gel electrophoresis of the urease gene PCR amplicon. *Left lane* approximately 329-bp fragment, *right lane* pUC19 DNA/MspI marker (Fermentas)

Secondly, urease gene fragment was amplified by PCR with the following ingredients: 25 pmol of TrURE1S and TrURE1R primers each [7], 0.5 µl of FastStart Taq DNA Polymerase (2.5 U, Roche), 10 µl of 5 × GC-rich solution, 5 µl of 10 × PCR buffer with 20 mM MgCl_2_, 1 µl of dNTPs (10 mM each), 0.5 µl of DMSO, 5 ng of DNA sample, all in a volume of 50 µl. Touchdown PCR was performed with the following program: pre-denaturation—4 min, 95 °C; amplification—35 × [denaturation: 1 min, 95 °C; annealing 1 min, 69–63 °C, step size 0.5 °C, step delay 0; elongation: 1 min, 72 °C]; cooling—1 min, 40 °C. Gel electrophoresis revealed that the product was approximately 330 bp long, suggesting a urease-positive *T*. *rubrum* rather than *T*. *rubrum* var. *raubitschekii* (Fig. 8). According to Hiruma et al. [7], the genomic sequence of a *raubitschekii* urease amplicon should also include a 68-bp intron and a 9-bp-long coding insertion (DDBJ AB719058, CBS 100084). The PCR product was sequenced from both ends. This analysis revealed a sequence of 329 bp (including primers) presenting a 100 % coverage with XM_003233873 (*T*. *rubrum* CBS 118892 urease (TERG_05790) mRNA) and with the corresponding genomic sequence (NW_003456423.1, Gene ID: 10376003). Both the 68-bp intron and the 9-bp insertion, specific to the CBS 100084 *raubitschekii* strain, were missing. Based on these results, it is more likely that the isolate was, in fact, a urease-positive *T*. *rubrum.*

## Discussion

*Trichophyton rubrum* var. *raubitschekii* is an anthropophilic, pathogenic dermatophyte, rarely found in Europe. Initially, it had been described as a separate species—*Trichophyton raubitschekii* [5, 9], yet molecular studies revealed it to be a variant of *T*. *rubrum* [4, 8]. It was described to differ from other *T*. *rubrum* variants by a limited growth, reddish brown color, the presence of numerous macro- and microconidia and the ability to produce urease [5, 8, 10]. The dermatophyte isolated at the clinic demonstrated all of these characteristics. Noteworthy, the Polish taxonomic key in operation states that the urease activity is the criterion for differentiation between *T*. *rubrum* var. *raubitschekii* and *T*. *rubrum* [11]. The characterization of urease-positive *T*. *rubrum* that are distinct from *T*. *rubrum* var. *raubitschekii* has clearly complicated the diagnostic process [2, 7].

The vast majority of *T*. *rubrum* var. *raubitschekii* cases originate in Asia, Africa and South America [1, 2, 12]. The aforementioned Polish patient had not traveled to these regions of the world. Furthermore, he did not claim contacts with anyone traveling to these regions, nor with people showing lesions that might be mycotic.

There has been a *T*. *rubrum* var. *raubitschekii* case of tinea faciei in Germany in a female patient born in Angola [12]. Furthermore, Brasch et al. [13] described a different German case, involving a 32-year-old male Cameroonian student with well distinct, erythematous, exfoliating lesions located on the abdomen as well as on both hands. The clinical picture suggested tinea manuum. A culture grown on Sabouraud medium at 26 °C and a urease test led to the diagnosis of *T*. *rubrum* var. *raubitschekii.* Both the Cameroonian and the described Polish patients responded well to their oral itraconazole treatments. To date, there have been eight described cases in Central Europe. All of them were Africans residing in Germany [3, 12–14]. The described Polish case is exceptional, as it appears not to be linked with Africa in any way.

Arabatzis et al. [15] described a study involving a group of patients from southern Europe. Cultures and urease tests were performed on samples originating from different parts of the skin of 95 Greek and 10 Bulgarian patients. *T*. *rubrum* var. *raubitschekii* was identified in five Greeks and one Bulgarian. The pathogen originated from lesions of tinea manuum (one case), tinea corporis (one case), tinea cruris (one case) and tinea unguium (three cases). The group originated from and resided in Greece and Bulgaria at the time of the study. They all had not traveled to the aforementioned endemic regions nor abroad for the past 6 months.

The molecular characterization of the urease gene of *T*. *rubrum* var. *raubitschekii* will probably become the method of choice for distinguishing between the quite alike urease-positive *T*. *rubrum* and *T*. *rubrum* var. *raubitschekii*. The procedure is straightforward and can easily be modified for probe or melting curve assays. For the time being, it has to be approached with caution as only one *T*. *rubrum* var. *raubitschekii* isolate (CBS 100084) has been analyzed in this way so far [7]. At the moment, public whole genome sequencing projects include multiple *T*. *rubrum* and two *T*. *rubrum* var. *raubitschekii* isolates: CBS 100084 and CBS 202.88. The results of these projects may answer the question whether the urease allele of the CBS 100084 strain is the specific marker of all *T*. *rubrum* var. *raubitschekii* isolates, as suggested by Hiruma et al. In the meantime, we encourage other researchers to reassess their *T*. *rubrum* var. *raubitschekii* isolates with this molecular approach.

We have described a possible case of an infection with *T*. *rubrum* var. *raubitschekii* in Poland. Irrespective of whether it was truly a *T*. *rubrum* var. *raubitschekii* or a *urease*-*positive* *T*. *rubrum,* there seems to be a necessity of including both into the Polish and other Central European diagnostic schemes, as their identification obviously poses a challenge at the moment. Moreover, it is noteworthy that the histopathological examination turned out to be very useful, if not necessary, in the diagnostic process. It enabled the adequate treatment to be implemented even before the results of the mycological cultures and molecular studies were available.
